# Impact of drying methods on natural antioxidants, phenols and flavanones of immature dropped *Citrus sinensis* L. Osbeck fruits

**DOI:** 10.1038/s41598-022-10661-7

**Published:** 2022-04-23

**Authors:** Dinesh Kumar, M. S. Ladaniya, Manju Gurjar, Sunil Kumar

**Affiliations:** ICAR-Central Citrus Research Institute, Nagpur, Maharashtra India

**Keywords:** Biochemistry, Plant sciences

## Abstract

Citrus fruits are famous for nutritional value and studies are there for extraction of secondary metabolites from citrus waste. An attempt was made to quantify antioxidants, flavonoids and phenols from dropped fruits of 8–24 mm size, to find the impact of freeze and hot-air oven drying techniques on extraction. Flavonoids (hesperidin, narirutin/isonaringin, diosmin and didymin/neoponcirin) were quantified through high performance liquid chromatography (HPLC) and total phenols (TPC) were estimated by Folin-Ciocalteu method. Antioxidant capacity was adjudged by azino-bis [3-ethylbenzthiazoline-6-sulfonic acid] (ABTS), 2, 2-diphenyl-1-picrylhydrazyl radical (DPPH) and Ferric Reducing Antioxidant Power (FRAP). Freeze dried fruits of 10 mm and 12 mm retained maximum hesperidin content (22.383% and 21.560%) in comparison to hot-air oven counterparts (18.377% and 15.090%). Narirutin/isonaringin (1.343% and 1.191%), diosmin (5.293% and 3.234%) and didymin/neoponcirin (1.187% and 1.113%) content were found higher in 8 mm and 10 mm freeze dried fruits. The antioxidant capacity (7.548–11.643 mmol L^−1^ Trolox, 8.164–14.710 mmol L^−1^ Trolox, 4.008–5.863 mmol L^−1^ Trolox by ABTS, DPPH and FRAP assays) and TPC were found higher in freeze dried samples. Significant correlation was found between antioxidant capacity, TPC and flavonoids at p < 0.01. Freeze drying technique can be adopted for retaining and quality extraction of bioactive compounds from immature dropped fruits for further use in nutraceutical industries.

## Introduction

Citrus fruits are among one of the major fruit crops and play an important role in contributing to the country’s economy^1,2^. The genus citrus belongs to the family of *Rutaceae* and encompasses major group’s namely *Citrus reticulata*, *Citrus sinensis*, *Citrus* *limon*, *Citrus aurantium*, *Citrus paradisi* and *Citrus grandis*^3,4^. Citrus fruits are cultivated and cherished all over the world due to its nutritional content and characteristic flavor, taste and aroma^5^. The consumption of fruits is directly related in prevention of diseases due to the presence of many bioactive compounds and phytochemcials. The health benefits are related mainly due anti-inflammatory, anti-oxidant, anti-fungal, anti-carcinogenic and cardio-protective activities inherent in fruits^6^. Citrus fruits are predominantly found to be the rich source of phytochemical like ascorbic acid, flavonoids, phenolics, antioxidants, carotenoids, etc. which plays a pivotal role in combating many diseases and development of nutraceutical and pharmaceutical drugs^3,4^.

Plants produce several different secondary metabolites and flavonoid is one of the metabolite predominantly present in citrus fruits. Citrus fruits contain wide variety of compounds all of which have been potentially divided into three major classes: flavones, flavanones and flavonols^7,8^. Chemically, flavonoids contain a characteristic C6-C3-C6 skeleton and are derived from 2-phenylchromone parent compound consisting of three different rings with varying degree of methoxylation and hydroxylation. The factors like degree of polymerization, functional groups like methyl, hydroxyl, oxygen, and other substitutions determines the class of flavonoids^9^. The glycoside and aglycone are two forms of citrus flavanones. In them, Naringenin and hesperitin comes under the aglycone forms, neohesperidosides (Naringin, neohesperidin and neoeriocitrin) and rutinosides (hesperidin, narirutin and didymin) comes under glycoside forms^10^. The characteristic flavonoid compound predominantly present in young citrus tissues is the hesperidin flavanone glycoside. It consists of hesperitin and sucrose (glucose and rhamnose) named as rutinose^11^. The concentration of flavonoid varies depending on genetic and environmental factors and is considered as unique fingerprint for each variety. The accumulation occurs during each developmental stage of fruit^12^. During normal metabolism, different reactive oxygen species (ROS) are formed which are harmful and cause damage to the human body^4^. Phenolic compounds occurring naturally in citrus fruits possess antioxidant capacity.

During citrus fruit growth and development, immature fruits drop from stem-branch or ovary-stem junctions which are still green in color due to physiological reasons. This is natural phenomenon which differs from that occurs due to storm, insect pests and diseases. Some of the reasons for physiological dropping of citrus fruits might be imperfect pollination, ovule dysplasia, degeneration; nutrient deficiency. Citrus fruits experience remarkable quantity of physiological dropping of fruits^13^. Since ancient times, drying technique is practiced in various food processing techniques and storage to extend the shelf life. Drying processes minimizes the moisture content and the associated harmful chemical reactions and thus prevents the growth of spoilage micro-organisms. Drying technique reduces the packaging, storage and transportation costs^14^. Dried fruits can be relished during off-season too. Many different drying methods have practiced by many researchers till date^15,16^. The traditional employed sun drying technique is used frequently for drying purpose due to low cost investment but has several disadvantages like long drying time, contamination by dust, insects, etc., weather dependency, low product quality^17^. Hence, in order to improve shelf life and to preserve the nutritional content of the agricultural produce it is necessary to explore more efficient drying processes^18^. Hot air oven drying technique can be controlled easily and convenient for use at laboratory scale. Oven drying is affordable and the temperature conditions can be monitored easily. The technique is also available during off-season. The nutritional content and sensory attributes of plant materials is retained due to the advantage of low temperature and pressure being used in vacuum freeze drying technique^19^. The effect of drying varies with different plant materials and compounds and is difficult to predict. Much information can be gathered about the various bioactive with reference to drying techniques^16^. Many studies have been conducted for profiling of phytochemical and antioxidant potential in various fruits juices and extraction techniques but limited knowledge is present in case of effect of drying methods employed for physiologically dropped citrus fruits.

In the present scenario, a dire interest in research on citrus flavonoids and antioxidants, this can extend the pool of the phyto-nutrients for humans. Taking into account the lack of information and relevance of dropped citrus fruits, an attempt was carried out to evaluate the impact of drying methods (freeze drying and hot air oven drying) on natural antioxidants, phenols and flavanones of immature *Citrus sinensis* L. Osbeck fruits. The results obtained and the information generated will serve as guideline for effective extraction of bioactive compounds. Further, the study will definitely confer further impulse to the application of small dropped citrus fruits usually considered as waste.

## Materials and methods

### Raw material

**Figure 1:**
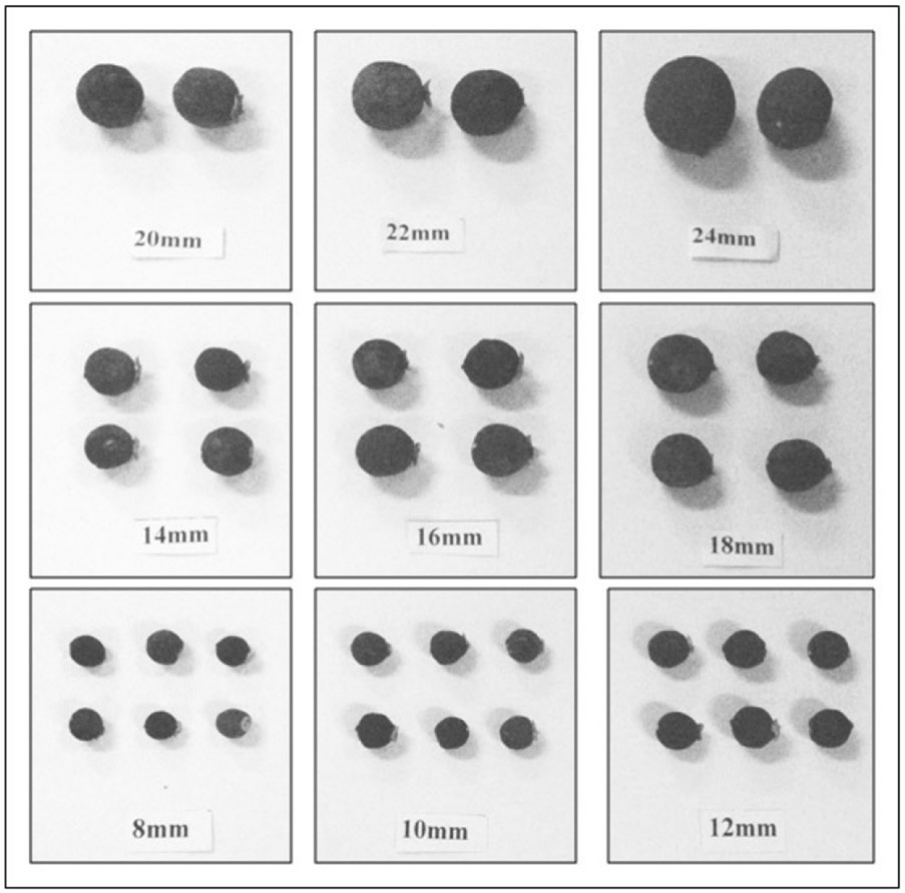
Dropped fruits of *Citrus sinensis* L. Osbeck (size 8–24 mm).

The study was carried out with immature dropped fruits of *Citrus sinensis* L. Osbeck from 8 to 24 mm diameter size collected during different developmental stages from the experimental block of ICAR-Central Citrus Research Institute in Nagpur (Maharashtra) (Fig. 1). In this experimental study, samples of immature dropped fruits of *Citrus sinensis* L. Osbeck were commonly found due to phenomenon of physiological dropping. Formal identification of the fruits analyzed in this study was not performed since it is a widely cultivated commercial variety grown from last 50 years and not newly identified variety. Permission of sample collection was gained in accordance with all the relevant institutional guidelines and legislation. The use of plants in the present study complies with international, national and/or institutional guidelines.

**Figure 2:**
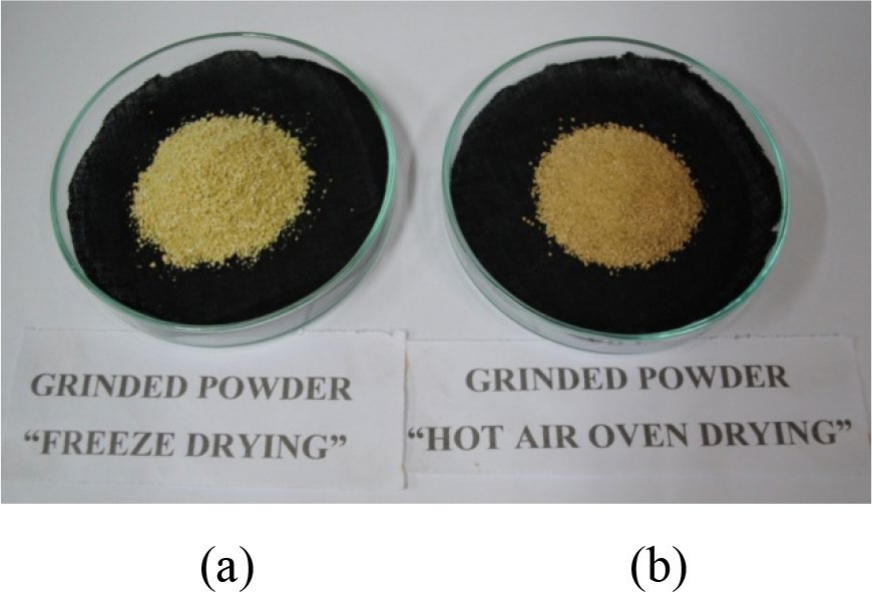
Grinded powder of dropped fruits of *Citrus sinensis* L. Osbeck—(**a**) After freeze drying and (**b**) After hot air oven drying.

The fruits were washed, cut into slices (0.5 cm thick) and used further for freeze drying and hot air oven drying. During hot air oven drying process, the sliced fruits were kept for 24 h to 36 h in microwave-oven (RIVOTEK, Riviera Glass Pvt. Ltd., Mumbai, India) set at temperature of 45–50 °C and in freeze drying technique, fruits were first kept in deep freezer (NEW BRUNSWICK™, Eppendorf, India) at − 20 °C for 12 h before lyophilization in vacuum freeze dryer (Mini Lyotrap, LTE Scientific Ltd., India) for 24 h to 48 h at − 50 °C to − 55 °C. The process of hot air drying and freeze drying is carried till complete drying of samples. The samples were finely grinded in mortar and pestle and passed through sieve of 50 micron to obtain uniform particle size powder (Fig. 2). The samples were stored in airtight polythene zip-lock pouches in the dark at – 20 °C till further use^4,13^.

### Chemicals

**Figure 3:**
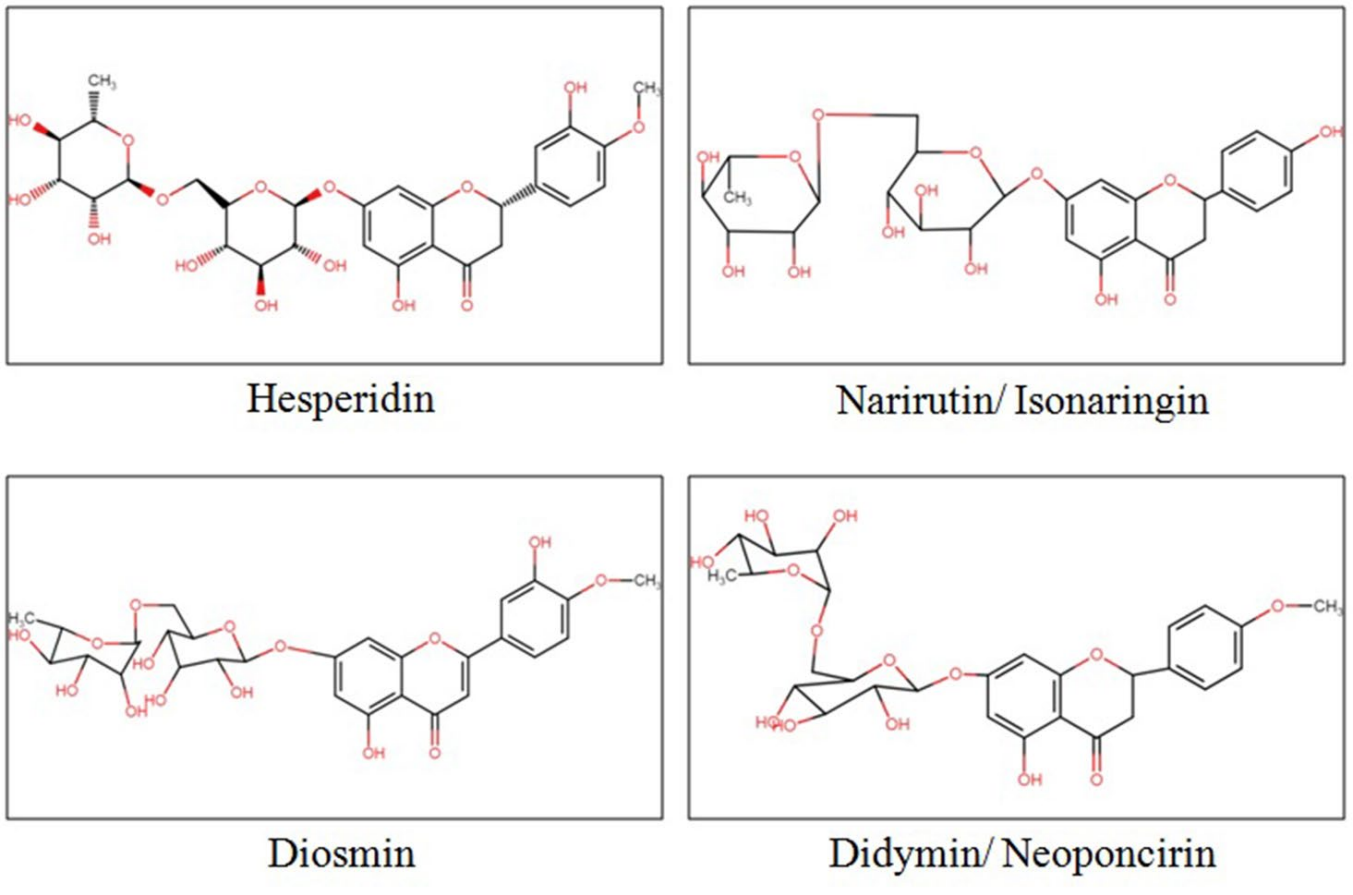
Chemical structure of citrus flavonoids (ChemIDPlus database)^20^.

Commercially available standards of flavonoids (hesperidin, narirutin/isonaringin, diosmin and didymin/neoponcirin) (Fig. 3), antioxidants i.e. ABTS^+^ (radical cation azino-bis [3-ethylbenzthiazoline-6-sulfonic acid]), 2, 2-diphenyl-1-picrylhydrazyl radical (DPPH), 2, 4, 6-Tri (2-pyridyl)-s-triazine (TPTZ), and gallic acid were from Sigma–Aldrich (Mumbai, India). HPLC grade chemicals of ammonium acetate, acetonitrile, dimethyl sulphoxide were used in chromatography method analysis and in extraction process. Other chemicals used were of AR grade.

### Apparatus and instruments

The antioxidant capacity was assessed with 96 well plates in an automated microplate reader Tecan Infinite M200 Pro (Tecan Group Ltd, Switzerland). High performance liquid chromatography was performed using reverse phase column, Nucleosil 100-C18, 4.6 mm, 100 mm length in HPLC Agilent Model No. 1260 Infinity System (M/s. Agilent Technologies Pvt. Ltd., Bangalore, India) for flavonoid quantification. Sonicator 2K1008008 series (Life-Care Equipments Pvt. Ltd., Mumbai, India) was used for sonication during sample preparation.

### Extraction and determination of flavonoids

A 3 mg of fruit powder was extracted in 5 mL of Dimethyl sulphoxide (DMSO) in a sonicator for 20 min. The solution was filtered through nylon filter (mesh size 0.45 micron) and injected to HPLC for measurement of flavanone glycosides^11^. The content was determined using reverse phase column, Nucleosil 100-C18, 4.6 mm, 100 mm length. The gradient mobile phase consisted of 5 mM ammonium acetate along with acetonitrile in 75:25 (v/v) ratio was used with acetic acid for pH adjustment. The sample and standards injection volume was 5µL and the temperature of column was 40 °C. The flavonoids were detected at wavelength of 284 nm respectively and recovery percentage was done by spiking fixed amounts of standards samples (600 ppm) of each flavonoids namely hesperidin, narirutin/ isonaringin, diosmin and didymin/neoponcirin and diluted using the mobile phase if required for calibration purpose^21^.

### Determination of antioxidant capacity

The method of ABTS^+^ (radical cation azino-bis [3-ethylbenzthiazoline-6-sulfonic acid]) (ABTS), 2, 2-diphenyl-1-picrylhydrazyl radical (DPPH) and Ferric Reducing Antioxidant Power (FRAP) were used to determine the antioxidant capacity. The assay of ABTS and DPPH was carried according to the literature procedure described by Mena et al.^22^. The reaction kinetics was monitored for about 50 min at 25 °C and absorbance was set to 414 nm in ABTS assay and 515 nm in DPPH assay. The trolox standard solution was used to construct the calibration curves. The antioxidant capacity was assessed by quenching the ABTS^+^ relative to trolox and due to the electron donation ability being measured by bleaching the DPPH purple colored solution. The assay of FRAP was performed according to a minor modification of the method as described by Benzie and Strain^23^. The reagent of FRAP used in the analysis was prepared freshly. Acetate buffer (300 mM), TPTZ and ferric chloride solutions used to prepare the FRAP reagent were added in the ratio of 10:1:1. The reaction kinetics was measured for 40 min at 25 °C at 593 nm. The antioxidant compounds present in the sample reduces the Fe (III)–tripyridyltriazine complex to the blue ferrous form. Calibration curve was prepared using trolox as standard and antioxidant capacity measured. The tests were carried out in triplicates. The results of antioxidant capacity assays are expressed in mmol L^−1^ Trolox.

### Determination of total phenolic content (TPC)

The total phenolic content was determined by Folin–Ciocalteu method described by Singleton and Rossi^24^. For quantification, 10 µL samples were added to 790 µL milli-Q water and 50 µL Folin–Ciocalteu reagents. The solution was vortexed and 150 µL of 20% sodium carbonate solution was mixed and after 60 min incubation time at room temp 23.5 °C in dark conditions, the absorbance was recorded at 750 nm. The blank consisted of all the chemicals and reagent solutions without the sample being added. The standard of gallic acid was used to prepare the calibration curve and the results were reported in mg GAE L^−1^.

### Statistical analysis

All the experiment data obtained was analyzed in three replicated trials and the data were expressed in the form of mean ± standard deviation. One-way analysis of variance (ANOVA) along with multiple range test (Tukey’s HSD) was used to analyze and determine the significance of the data obtained. Pearson correlation analysis was carried out to assess the correlation among the parameters^25^. The parameters at probability values (p) of < 0.01 was regarded as a significant correlation.

## Results and discussion

### Effect on flavonoids content

Flavonoids in immature fruits are one of the natural antioxidant sources available and are useful in prevention of diseases^26,27^. Quantification of flavonoids in dropped fruits of *Citrus sinensis* varying in size from 8 to 24 mm were assessed using high performance liquid chromatography (HPLC). HPLC chromatograms of four different flavanone glycosides viz. hesperidin, narirutin/isonaringin, diosmin and didymin/neoponcirin were quantified according to the retention time and their peaks against those of standards at wavelength of 284 nm. Table 1 gives the flavonoid content in relative percentage. To evaluate the effect drying technique on each flavonoid concentration, the mean values obtained were compared statistically using the Tukey’s test and the data obtained was also found to be significant at p < 0.01. It is evident from the table that the flavonoids content responded considerably with respect to the drying technique. Among the flavonoids assessed, hesperidin was the most abundantly present. The hesperidin content obtained from 8.597 to 22.383% in freeze dried samples and from 6.120 to 18.377% in hot air oven dried samples. Maximum relative percentage of hesperidin was obtained in immature fruits i.e. from 8 to 14 mm size with 20.087%, 22.383%, 21.560% and 20.223% respectively by freeze drying technique. A slight decreased was noticed in hot air oven dried samples. According to Hirsch^28^, activation of oxidative enzymes like polyphenol oxidase during hot air oven drying leads to the loss of flavonoids content. The activity of polyphenol oxidase enzyme was decreased in freeze drying operated at the lower temperature. Kim and Kim^2^ also reported the hesperidin as the widely distributed flavonoid in thinned immature *Citrus unshui* fruits. Similar results were also documented by several researchers^13,29^ while carrying out study with young mandarin fruits, physiologically dropped immature citrus fruits.

**Table 1:** Flavonoids profile (relative %) after different drying techniques (freeze drying for about 48 h and hot air oven drying for about 36 h) in *C. sinensis* L. Osbeck immature dropped fruits.

Sr. no.	Fruit size (mm)	Drying methods	Hesperidin (%)	Narirutin/isonaringin (%)	Diosmin (%)	Didymin/neoponcirin (%)
1	8	Freeze drying	20.087^c^ ± 0.45	1.343^a^ ± 0.07	5.293^a^ ± 0.25	1.187^a^ ± 0.18
		Hot air oven drying	16.443^b^ ± 0.39	1.096^a^ ± 0.04	nd	1.070^a^ ± 0.09
2	10	Freeze drying	22.383^a^ ± 0.44	1.191^ab^ ± 0.03	3.234^b^ ± 0.04	1.113^ab^ ± 0.02
		Hot air oven drying	18.377^a^ ± 0.16	0.963^ab^ ± 0.02	nd	0.897^ab^ ± 0.08
3	12	Freeze drying	21.560^ab^ ± 0.46	1.092^bc^ ± 0.03	2.593^c^ ± 0.24	1.004^ab^ ± 0.02
		Hot air oven drying	15.090^c^ ± 0.14	0.900^bc^ ± 0.03	nd	0.817^bc^ ± 0.07
4	14	Freeze drying	20.223^bc^ ± 0.54	1.006^bc^ ± 0.02	0.657^d^ ± 0.04	0.952^ab^ ± 0.02
		Hot air oven drying	14.010^d^ ± 0.14	0.827^bcd^ ± 0.02	nd	0.753^bc^ ± 0.06
5	16	Freeze drying	18.233^d^ ± 0.62	0.887^c^ ± 0.02	0.517^d^ ± 0.07	0.913^ab^ ± 0.03
		Hot air oven drying	13.712^d^ ± 0.22	0.780^ cd^ ± 0.03	nd	0.737^bcd^ ± 0.05
6	18	Freeze drying	15.567^e^ ± 0.23	0.960^c^ ± 0.09	nd	0.988^ab^ ± 0.13
		Hot air oven drying	12.850^e^ ± 0.22	0.720^de^ ± 0.07	nd	0.663^ cd^ ± 0.03
7	20	Freeze drying	12.673f. ± 0.41	0.994^bc^ ± 0.03	nd	0.953^ab^ ± 0.07
		Hot air oven drying	10.090f. ± 0.18	0.690^de^ ± 0.02	nd	0.680^ cd^ ± 0.02
8	22	Freeze drying	10.251^ g^ ± 0.14	1.061^bc^ ± 0.09	nd	0.885^bc^ ± 0.03
		Hot air oven drying	8.390^ g^ ± 0.26	0.627^ef^ ± 0.03	nd	0.683^ cd^ ± 0.03
9	24	Freeze drying	8.597^ h^ ± 0.20	0.548^d^ ± 0.10	nd	0.610^c^ ± 0.03
		Hot air oven drying	6.120^ h^ ± 0.10	0.493f. ± 0.09	nd	0.547^d^ ± 0.04
Tukey’s HSD at 1%	Freeze drying	1.4638	0.2244	0.4185	0.2777	
	Hot air oven drying	0.7583	0.1485	–	0.1910	

*nd* not detected. Data presented is analyzed in three replicated trials and expressed as means ± standard deviation. Values with different superscripts in a column are significantly different at p < 0.01 according to the Tukey’s honestly significant difference (HSD) multiple range test, Values with same superscripts are not significantly different.

**Figure 4:**
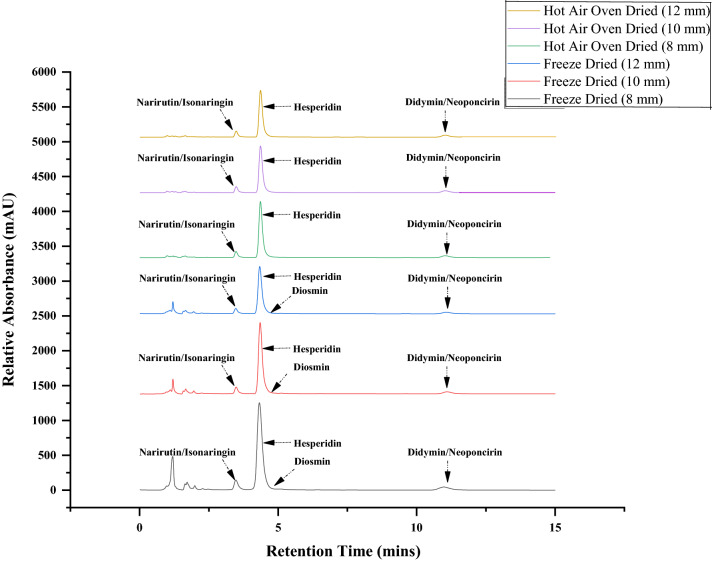
HPLC chromatogram of flavonoids content of dropped immature fruits *C. sinensis* L. Osbeck varies from size 8 mm, 10 mm and 12 mm after freeze drying and hot air oven drying recorded at wavelength 284 nm.

In case of flavonoid narirutin/isonaringin, maximum compounds were quantified in freeze dried samples (0.548–1.343%) followed by hot air oven dried samples (0.493–1.096%). Highest amount was quantified in immature fruits^11^ and the content decreased with developmental stage of fruit i.e. with increase in diameter. Sun et al.^13^ found that the narirutin content in citrus species of Ponkan and Gaocheng were more after freeze drying in comparison to the sun and hot air drying counterparts. There are several reports^30,31^ mentioning the higher content of hesperidin and naringin content in different cultivars of citrus (mandarin, orange, grapefruit, pummelo). The results are in agreement with study conducted by Ye et al.^29^ in young mandarin fruits and with Kumar et al.^4^ in dropped *Citrus reticulata* Blanco fruits. In a study with immature *Citrus unshui* pomace, the content of hesperidin and naringin was approximately 2.32–2.34 fold times higher in immature fruits than mature ones^32^. Similar reports were also obtained by Ortuno et al.^33^ in immature varieties of grapefruit and pummelo. Table 1 depicts the fairly high amount (relative percentage) of didymin/neoponcirin of freeze dried samples (0.610–1.187%) whereas, hot air oven dried samples demonstrated lower content (0.547–1.070%). Thermal method of hot air oven drying caused loss in content when compared with freeze drying. Any drying process which deteriorates the cellular structure leads to loss of flavanol compounds stored outside the organelle^34^. In our study conducted, flavonoid diosmin content was from 2.593 to 5.293% in immature fruits of size 8 mm, 10 mm and 12 mm fruits, in traces (0.517% and 0.657%) in 16 mm and 14 mm and was not found in fruits varying in size 18–24 mm respectively. However, interestingly the diosmin was not detected in hot air oven dried samples. In accordance to our results, Kumar et al.^4^ also could not identify the diosmin flavonoid in dropped fruits of *Citrus reticulata* Blanco after hot air drying technique. The estimation of diosmin should be carried out in detail by other researchers and warrants future studies. The chromatograms of 8 mm, 10 mm and 12 mm dropped fruits obtained through HPLC which retained maximum content of hesperidin (20.087–22.383%) and other flavonoids after freeze drying in comparison with recovery percentage obtained after the hot air drying technique are depicted in Fig. 4. Barecca et al.^12^ found the immature chinotto fruits to be rich in flavonoids than ripe fruits. Various factors like genetic and environmental factors namely climate, soil conditions, storage, etc. influences the amount and distribution of flavonoids in citrus cultivars^35^ and substantial quantity is accumulated during the juvenile stage of fruit development^36^.

Based on the results obtained and according to the studies carried out earlier by other researchers, freeze drying technique varied at low temperature under vacuum can be adopted for efficient extraction of flavonoids content from immature *Citrus sinensis* dropped fruits.

### Effects on antioxidant capacity assays

Free radicals are harmful and thus antioxidants scavenge these free radicals and play a very important role in preventing of diseases like cancer, diabetes, alzheimer, etc.^37^. The antioxidant capacity exerted by natural plant sources depends on two factors—the reaction mechanism and the distribution of antioxidant compounds in two phases viz. include hydrophilic phase and lipophilic phase. Hence, relying on only any one method for assessing the antioxidant capacity is not justifiable^38^. Antioxidant capacity was determined by more than one technique. Among many widely adopted methods, the assays of ABTS, DPPH and FRAP were employed in the study carried out.

**Figure 5:**
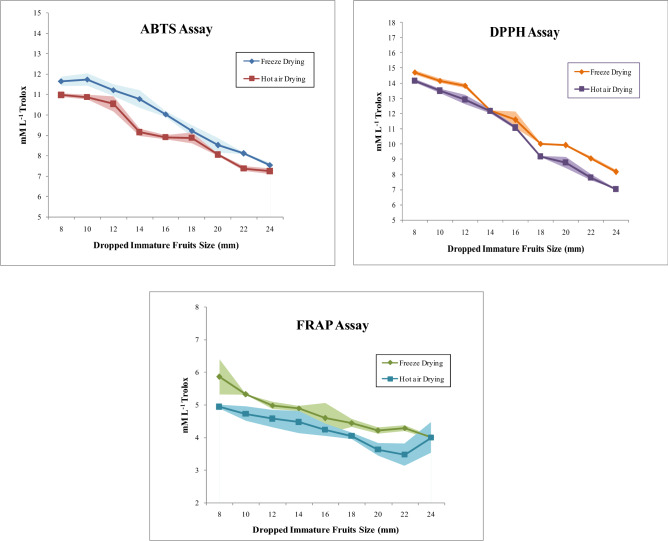
Antioxidant radical scavenging capacity by ABTS, DPPH and FRAP assays of freeze dried and hot air oven dried samples of immature *C. sinensis* L. Osbeck dropped fruits.

The ABTS assay is based on the scavenging of free radicals and conversion into the colorless product. More is the antioxidant capacity of sample, more is the degree of discolor action^39^. The DPPH is a stable free radical. The assay of DPPH is one of the widely accepted methods used to determine the antioxidant capacity^40^. The antioxidant capacity of samples determines the degree of discoloration. Results of antioxidant capacity assays of ABTS and DPPH are presented in Fig. 5. The capacity as measured by the ABTS assays ranged from 7.548–11.643 mmol L^−1^ Trolox in freeze dried samples and from 7.235 to 10.971 mmol L^−1^ Trolox in hot air oven dried samples whereas in DPPH assay, the antioxidant capacity ranged from 8.164 to 14.710 mmol L^−1^ Trolox in freeze dried samples and obtained from 7.025 to 14.172 mmol L^−1^ Trolox in hot air oven dried samples. Dropped immature fruits of *C. sinesis* L. Osbeck from 8 to 12 mm size had the highest antioxidant capacity as compared to the mature fruits. Kim and Kim^2^ also reported higher antioxidant capacity in thinned immature *Citrus unshiu* fruits. It was observed that physiologically dropped immature fruits dehydrated by freeze drying exhibited higher antioxidant capacity than the fruits dried by hot air drying technique. It reflects that the cellular structure gets disintegrated during the heating process leading to the oxidation of thermo-labile compounds sensitive to degradation. Sun et al.^13^ recommended the use of freeze drying technique to optimization of antioxidant capacity. Sim et al.^41^ while working with *Grifolia frondosa* also observed that oven drying leads to considerable loss in radical scavenging capacity in comparison to fresh mushroom. Similar type of findings were also reported by Cano and others^30^; Agudelo et al.^42^ and Kumar et al.^4^ while carrying study with immature Chinotto juice, freeze dried grapefruit fruits, different citrus varieties, small size immature kumquat and dropped *Citrus reticulata* Blanco fruits respectively. In FRAP assay, Fe (III) tripridyltriazine complex is reduced to blue ferrous from by the antioxidant present in the sample^23^. The antioxidant capacity of sample corresponds to the reducing capability to transfer electrons to reagent of FRAP^43^. The FRAP values varied from 4.008 to 5.863 mmol L^−1^ Trolox (Freeze drying) and 3.998–4.941 mmol L^−1^ Trolox (Hot air oven drying). As depicted in Fig. 5, 8 mm and 10 mm size dropped fruits contained higher antioxidant capacity. Physiological dropped fruits of citrus retained potential antioxidant capacity^29,31^. Among the drying methods employed, hot air oven technique showed the low radical scavenging capacity in comparative to freeze-drying technique. The technique of freeze drying retained various bioactive compounds present. Freeze drying technique is found satisfactory in radical scavenging ability^13^.

### Effects on total phenol content

**Figure 6:**
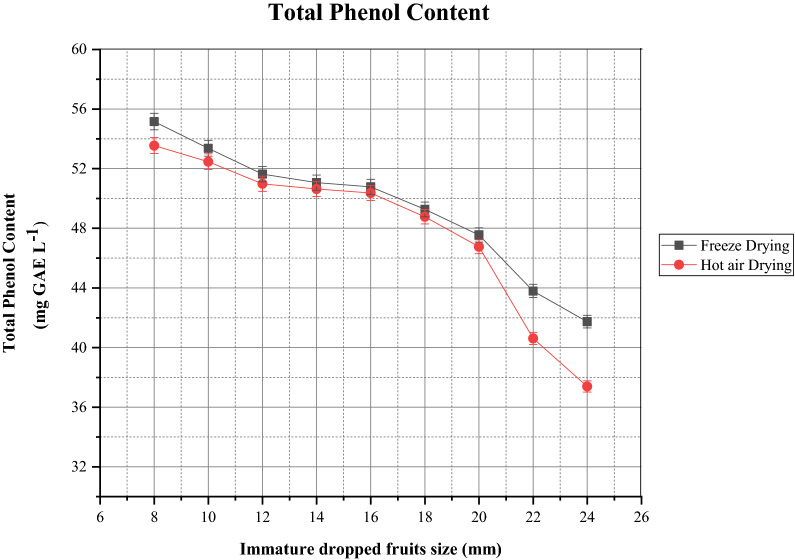
Content of total phenol (TPC) after freeze drying and hot air oven drying in different sizes of immature *C. sinensis* L. Osbeck dropped fruits.

Phenolic complexes play a key role in cells and inherit potential antioxidant capacity. Fruits containing higher total phenol content depict higher antioxidant capacity^2,31^. The amounts of total phenol in immature dropped fruits of *Citrus sinensis* were determined and the results are depicted in Fig. 6. The total phenol content ranged from 41.736 to 55.161 mg GAE L^−1^ after freeze drying. The hot air oven dried samples contained 37.393–53.548 mg GAE L^−1^ total phenol content. 8 mm and 10 mm size fruits were highest TPC content of 55.161 mg GAE L^−1^ and 53.370 mg GAE L^−1^ respectively and are good source of phenol compounds. Kim and Kim^2^ and Kumar et al.^4^ reported higher total phenol content in immature *Citrus unshiu* and dropped *Citrus reticulata* Blanco fruits than the mature fruit. We observed that freeze drying technique displayed higher phenol content and therefore the antioxidant capacity. Phenol compounds are sensitive to temperature^44^ and if the cellular structure is destroyed during drying technique, there is considerable loss in content. Mechanism of activation enzymes (polyphenoloxidase and peroxidase) leads to the loss of phenolic complexes during thermal drying process^15^. Besides, factors like changes in chemical structure, binding of phenols to protein also influences and responsible for the loss of phenolic content^45^. Similar kind of results was observed during studying the effect of drying on tomatoes and ginger by Gumusay et al.^15^. The results obtained in the study are in correlation with Ye et al.^29^ and Sun et al.^13^ who experimented with young mandarin and immature citrus fruits respectively. Cano et al.^30^ and Barecca et al.^12^ reported greater antioxidant capacity in immature citrus fruits with respect to mature fruits. From the results obtained, it can be inferred that maturity influences the concentration of total phenols. The higher phenolic content is estimated at early developmental stages of the fruit.

### The correlation coefficients of flavonoids and total phenols with antioxidants

**Figure 7:**
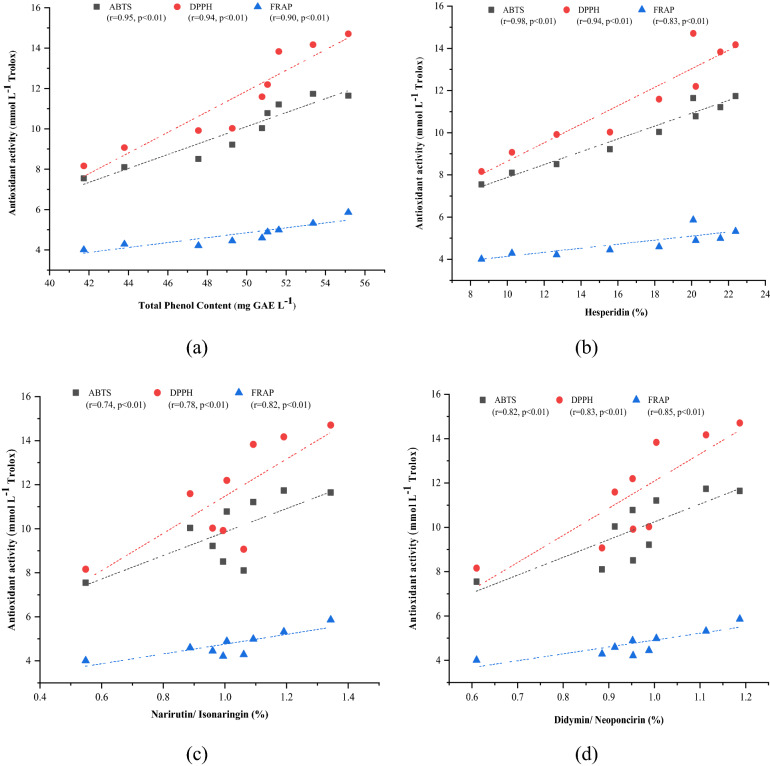
Graphs represent the correlation coefficient between—(**a**) antioxidant capacity and Total phenol, (**b**) antioxidant capacity and hesperidin flavonoid, (**c**) antioxidant capacity and narirutin/isonaringin flavonoid and (**d**) antioxidant capacityand didymin/neoponcirin flavonoid. The correlation coefficient values obtained were significant at p < 0.01.

Freeze drying being the effective method in comparison with hot air drying for extraction and quantification of flavonoids, phenol compounds and antioxidant capacity, a correlation was determined between the parameters assessed and results are depicted in Fig. 7. Flavonoids and phenolic compounds possess antioxidant activities, scavenges harmful reactive free oxygen species due to their ability to donate hydrogen atoms to the free radicals^46^. The antioxidant capacity from ABTS, DPPH and FRAP assays was very well correlated with the TPC (Fig. 7a) with correlation coefficients of 0.95, 0.94 and 0.90 respectively at p < 0.01. Thus, we can conclude that phenols have potential free radicals scavenging capacity. Many studies have also reported significant correlation between phenol compounds and antioxidants^4,31,47^. Antioxidant capacity is also displayed by flavonoids^31^. In the present study, Fig. 7b depicts positive correlation was also observed between hesperidin and antioxidants (r = 0.98 with ABTS assay, r = 0.94 with DPPH assay and r = 0.83 with FRAP assay) at p < 0.01 i.e. 1% level of significance. It is depicted in the Fig. 7c the flavonoid narirutin/isonaringin showed correlation with ABTS, DPPH and FRAP assays; the correlation coefficients being 0.74, 0.789 and 0.82 at p < 0.01. Hesperidin and narirutin are the major flavanone glycosides predominantly present in higher amounts in the immature citrus species^29,33,48^. The significant correlation was also obtained between didymin/neoponcirin and ABTS (r = 0.82 at p < 0.01), didymin/neoponcirin and DPPH (r = 0.83 at p < 0.01) and also between didymin/neoponcirin and FRAP (r = 0.85 at p < 0.01) antioxidant assays (Fig. 7d). Flavonoids in citrus immature fruits are one of the naturally available sources of antioxidants^27^. Hesperidin flavonoid showed a stronger correlation with the antioxidant assays employed in the study in comparison with narirutin/isonaringin and didymin/neoponcirin. The higher antioxidant capacity of hesperidin in comparison with others is attributed to the catechol group which is present in the B-ring structure of the hesperidin molecule^49^.

**Figure 8:**
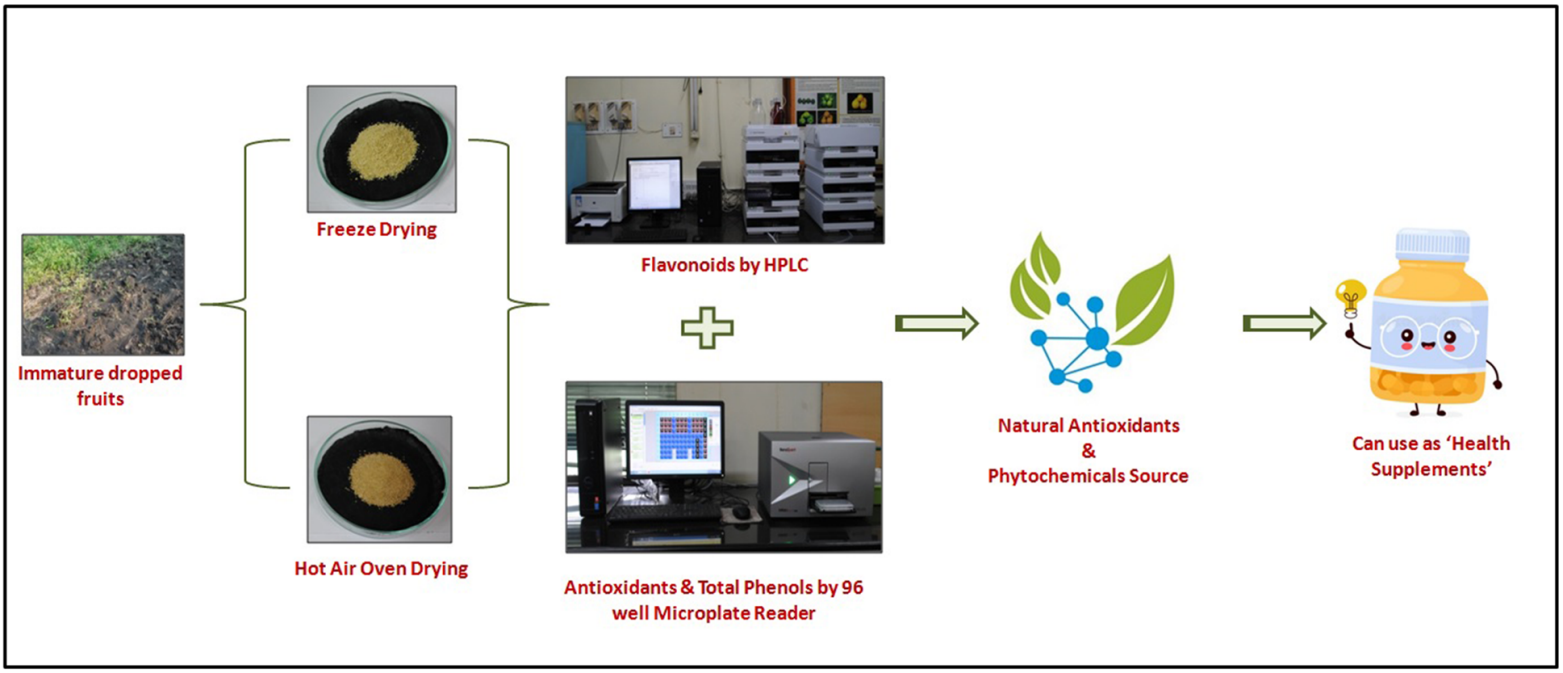
Graphical abstract of the study.

Figure 8 represents the graphical abstract of the study carried out. The results suggest that the immature dropped fruits of *C. sinesis* L. Osbeck can be considered as rich natural plant source for extraction of bioactive compounds and for its future applications in pharmaceutical and nutraceutical industry. Freeze drying technique can be recommended for optimizing and retaining these functional components and antioxidant capacity.

## Conclusions

The influence of drying methods (freeze drying and hot air oven drying) on bioactive compounds and antioxidant capacity in nine different sizes of immature dropped fruits of *Citrus sinensis* has been examined. It was found that the hesperidin is the major flavanone glycoside found in immature fruits. The greatest amount was found in 10 mm and 12 mm size fruits. The TPC and antioxidant capacity varied significantly by drying techniques and was retained through freeze drying method. The content increased as the maturity period is delayed. The technique of hot air drying caused loss in the prime nutritional content when compared with freeze drying. On the basis of correlation coefficients determined between bioactive compounds and antioxidants, clearly the largest contribution to the antioxidant capacity of physiologically dropped fruits are provided due to flavonoids and phenol which can effectively scavenge free radicals or reactive oxygen species in vitro conditions. In the present scenario, a dire interest in research on citrus flavonoids and antioxidants, this can extend the pool of the phyto-nutrients for humans. The result of the study indicates the use of dropped citrus fruits as potential sources of phytochemicals and natural antioxidants. Due to health consciousness of the people in the present era and their increase interest in naturally available food source, these antioxidant-rich dropped fruits will become as a valuable source for its use in food supplements after further processing. The advantages of the study is that dropped fruits considered as waste, can be utilized as a rich and potential source of bioactive compounds and flavanones i.e. hesperidin as a protective food supplement. Further, it will provide a platform for producing nutraceuticals which are efficient and eco-friendly. It is also going to play a vital role as the extracts can be used as a confectionary product for having the high-end value added product development.

## Data Availability

All data generated or analysed during this study are included in the article.
